# A Trust-Based Adaptive Probability Marking and Storage Traceback Scheme for WSNs

**DOI:** 10.3390/s16040451

**Published:** 2016-03-30

**Authors:** Anfeng Liu, Xiao Liu, Jun Long

**Affiliations:** School of Information Science and Engineering, Central South University, Changsha 410083, China; afengliu@mail.csu.edu.cn (A.L.); xiaoliu@csu.edu.cn (X.L.)

**Keywords:** traceback, wireless sensor network, trust, adaptive probability marking

## Abstract

Security is a pivotal issue for wireless sensor networks (WSNs), which are emerging as a promising platform that enables a wide range of military, scientific, industrial and commercial applications. Traceback, a key cyber-forensics technology, can play an important role in tracing and locating a malicious source to guarantee cybersecurity. In this work a trust-based adaptive probability marking and storage (TAPMS) traceback scheme is proposed to enhance security for WSNs. In a TAPMS scheme, the marking probability is adaptively adjusted according to the security requirements of the network and can substantially reduce the number of marking tuples and improve network lifetime. More importantly, a high trust node is selected to store marking tuples, which can avoid the problem of marking information being lost. Experimental results show that the total number of marking tuples can be reduced in a TAPMS scheme, thus improving network lifetime. At the same time, since the marking tuples are stored in high trust nodes, storage reliability can be guaranteed, and the traceback time can be reduced by more than 80%.

## 1. Introduction

Wireless sensor networks (WSNs), as one pivotal core components of the Internet of Things (IoT) [1,2,3,4], are emerging as a promising platform that enables a wide range of military [5], scientific [6], industrial and commercial applications, including monitoring of extended biological habitats, agriculture, industrial processes and human health-critical infrastructure [7,8,9,10]. However, sensor networks also face many security and privacy challenges due to the open nature of wireless communication in sensor networks and the limited capabilities of sensor nodes in terms of processing power, storage, bandwidth, and energy [9,10,11,12,13,14]. WSNs sense information with deployed sensor nodes and send the sensed information to a sink by multiple-hop paths [15]. In the process, the network might be subject to many attacks [16,17,18,19,20], for example, DDoS attacks, flood attacks, select forwarding attacks [16,17], injected false data attacks [18], and clone attacks [19]. Traceback is a security protection technology for WSNs [20,21,22,23,24]. One of the most important methods is packet marking [20,21,22,23], whose basic principle is that when the sensor nodes transmit data packets to the sink node, the sensor nodes add their ID information (called marking tuples) to the data packets. When the sink receives this information, it can reconstruct the path from the sink to the source nodes by analyzing these marking tuples. After the malicious source is determined, measures can be taken to block or remove the malicious sources to protect network security. Logging is also an effective traceback method [20,23,24]. In a logging scheme, when the number of marking tuples in the forwarded data packets reaches a predetermined threshold, those marking tuples can be stored in nodes. Therefore, the amount of data loaded by nodes near the sink can be reduced, and network lifetime can be improved. However, shortcomings still exist in the previous studies: (1) the marking probabilities of all nodes are the same and are determined in advance. Most nodes in the network are “good” nodes. The “good” nodes are usually considered credible, so those “good” nodes need not be marked, which can save energy; (2) the state of a node often changes; for example, if nodes are credible, the marking probability of nodes is low, but if nodes evaluated by the sink are untrusted, a high marking probability must be imposed for nodes to identify their status quickly. Therefore, in the process of network operation, the marking probability of nodes must be adjusted depending upon the extent to which nodes are trusted; (3) nodes are randomly selected to store marking tuples in previous schemes, so marking tuples could be stored in malicious nodes, which leads to the dropping of marking tuples which can prevent the malicious source node from being identified quickly. A better scheme should improve the energy efficiency, the method is that the system make full use of the remaining energy. The energy efficiency refers to the ratio of the used energy to the total energy. In order to guarantee better performance, the network lifetime should be improved, which is the time of rounds for operation. High network lifetime can ensure the reliability of a network, and it also can save energy for many applications [25,26]. Based on the above analysis, a trust-based adaptive probability marking and storage traceback (TAPMS) scheme is proposed to enhance security for WSNs. The main contributions of the TAPMS scheme are as follows:

(1) In the TAPMS scheme, the marking probability of nodes is adaptively adjusted according to their trust. First, in data transmissions, the times a network is attacked can be detected by the sink; thus, the security level in the network can be evaluated by the sink. The process for evaluating the security level is that before beginning data transmission, the sink provides predetermined attack times. At the time of data transmission, if the attack time of the network is greater than the predetermined value, the security level in the network has not improved. Therefore, the security level in the network requires adjustment. After time slots τ, the security level in the network can be evaluated by the sink according to the difference between two consecutive time slots. If the attack time in this time slot is less than the attack times in the last time slot, the security level has become good, therefore, the security level of the network will be better in the next time slot τ. It is reasonable to set a low marking probability in a secure network to save energy, but to set a high marking probability in an insecure network to locate the source(s) of malicious nodes. Therefore, the marking probability can be set in the TAPMS scheme as follows: if the network is in a “safe” state, the baseline of the marking probability can be set low to reduce the number of marking tuples. In contrast, the baseline of the marking probability can be set high when the security level of the network is low. Second, if node trust is high, the marking probability of nodes can be reduced. Conversely, the marking probability of nodes with low trust should be high. Because most nodes are marked with low probability, it will be easy to determine that the average marking probability of nodes in TAPMS is lower, the traceback time is shorter, and network lifetime is longer.
(2) In the TAPMS scheme, marking tuples are stored in high trust nodes to ensure stored marking tuples with high reliability. In previous schemes, nodes are randomly selected to store marking tuples. If marking tuples are stored in low trust nodes, those marking tuples can be dropped easily, which leads to a loss of marking tuples that are used to reconstruct the path from the sink to source nodes; therefore, the performance of the scheme is poor. In the TAPMS scheme, marking tuples are stored in high trust nodes, thus, the performance of this scheme has been improved.
(3) The theoretical analysis and experimental analysis demonstrate that traceback time and lifetime in TAPMS are both improved. The results show that the number of total marking tuples can be reduced by 13.20%–73.70% in the TAPMS scheme compared with that in the probability marking (PM) scheme with marking probability equal to 1 to improve network lifetime, and the traceback time can be reduced by more than 80%.

The remainder of this paper is organized as follows: in Section 2, the related work is reviewed. The system model is described in Section 3. In Section 4, the details of a trust-based adaptive probability marking and storage traceback (TAPMS) scheme are presented. Section 5 comprises the analysis and comparison of experimental results, and Section 6 presents the conclusion.

## 2. Related Work

Packet marking is an effective and popular traceback scheme used in wireless sensor networks (WSNs) [20,21,22,23,24]. In a packet-marking scheme, when the sensor nodes forward packets, each node adds its ID and other information (*i.e.*, marking tuples) to packets. After the sink receives the data packets, the sink node can reconstruct the path to the source nodes by reading marking tuples [21,22,23]. If the source node is a malicious node, the system will block or isolate the malicious node. The advantages of this scheme are that it is a simple protocol and almost no storage space is required for the nodes, so it is easy to implement. The disadvantages are that the number of marking tuples will grow with the number of packets forwarded to the sink. Therefore, the data packets might require division into many pieces to be sent to the sink, which not only increases routing conflicts but also reduces network lifetime [20,24]. To diminish the effect of marking on network lifetime, the probability marking method (PM) was proposed in [23]; each node marks data packets with a certain probability, which can reduce the amount of marking and increase the traceback time.

The scheme based on log (logging) is another type of tracking technology for malicious nodes [23]. In this scheme, when the marking field in data packets is large, the marking information is stored in the node memory; then, the nodes forward packets with unloading marking information to the next node. In the traceback process, the sink rebuilds a path from the sink to the source node through querying marking information stored in those nodes. Thus, the scheme based on logging can greatly reduce the amount of data received by the sink.

In [25] a combined packet marking and logging scheme for traceback (CPMLT) scheme, which combines marking and logging, was proposed. In the CPMLT scheme, a data packet can be marked at most k times, with each node marking a data packet with a certain probability; the nodes log the data packet after it has been marked k times.

Liu, *et al.* [20] also proposes a Logging joint Migrating (LM) traceback scheme. The most important improvement in the LM scheme compared with previous schemes addresses the issue that in previous traceback schemes, the energy consumption and storage space of nodes near the sink area are too high and seriously insufficient, respectively, with much storage capacity and energy left in areas far from the sink area. In the LM scheme, the packets are also marked at most k times, and each packet starts logging after been marked k times. When a node’s storage space is not sufficient, the data packet will be migrated to an area far from the sink, a strategy that can improve the network lifetime. Therefore, the scheme can make full use of residual energy and storage space.

Serra, *et al.* [27] proposed an energy scheduler and an energy scheduling method. In this energy scheduler, the scheme minimizes the energy consumption cost for a particular time interval, taking into account the energy price and a set of comfort constraints. The scheme can improve both the energy consumption and cost.

## 3. System Model

### 3.1. Network Model

**Figure 1:**
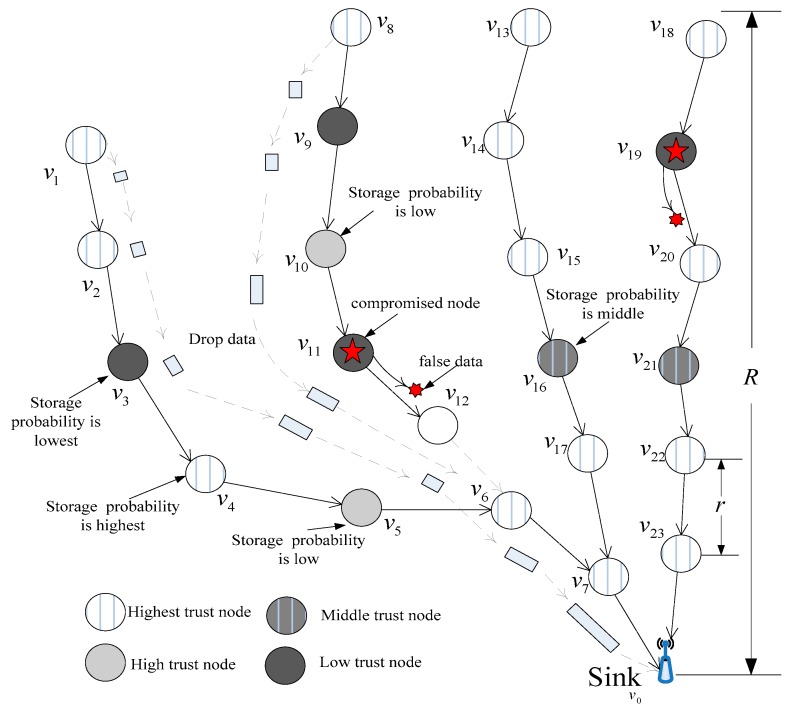
Network model.

(1) We consider a WSN consisting of m homogeneous static sensor nodes $v_{i}|i\in \left\{1..m\right\}$ and Sink node $v_{0}$, m nodes deployed over a 2-D circular surveillance field, and a network radius of R. Sink node $v_{0}$ is the center of the network. The communication radius of each sensor node is r. The network model is shown in Figure 1. The nodes have different trust levels, where most nodes are high trust nodes and a few nodes are low trust nodes. The node $v_{11}$ and node $v_{19}$ are compromised nodes with low trust in Figure 1. If the marking tuples are stored in those nodes, the marking tuples would be dropped with high probability. The marking tuples are dropped with low probability if the node trust level is high. The energy of each sensor node is limited, and the energy of the sink is infinite. Sensor nodes monitor their surroundings, and once an event is generated, nodes report to the base station through multi-hop transmissions [12,13].
(2) We consider the following attack scenario: a compromised node used to launch a false data injection attack to exhaust network resources is designated as the attack or source node [20,23,24,28]. Nodes mark packets with a certain probability P; in the event of an attack, the system can locate a malicious sources through those information marks, which is similar to cyber-forensics technologies [20,21,22,23,24,28].
(3) The sink can assess the trust of each node based on the marking tuples.

### 3.2. Energy Consumption Model and Related Definitions

The typical energy consumption model is adopted [29,30,31,32,33], as reflected in the energy consumption for sending data in Equation (1) and for receiving data in Equation (2):

(1) $$\left\{ \begin{array}{l} E_{t}=lE_{elec}+l\varepsilon_{fs}d^{2}\;if\;d<d_{0}\\ E_{t}=lE_{elec}+l\varepsilon_{amp}d^{4}\;if\;d>d_{0} \end{array}\right.$$

(2) $$E_{r}(l)=lE_{elec}$$

*E_elec_* in the formula represents the energy consumption per bit. If the transmission distance is less than the threshold *d*_0_, the consumption of power amplification adopts the free space model. If the transmisson distance is greater than the threshold *d*_0_, the power amplification consumption adopts the multipath attenuation model. ε*_fs_* and ε*_amp_* represent the energy required to amplify power in the two models. *l* denotes the number of bits of data.

In this paper, the parameters of the specific configuration in references [31,33,34] are shown in Table 1.

### 3.3. Problem Statement

The focus of this paper is to design a new, effective TAPMS scheme to trace back all types of attacks in WSNs. The goal of the TAPMS scheme is to locate the malicious source(s) as soon as possible at lower cost, which can be categorized by the following aspects:

(1) Network lifetime Γ is to be maximized.
  The basic goal of this application requirement is to maximize network lifetime. Network lifetime can be defined as the elapsed time until the first node dies [7,14,33,34]. The death of the first node can affect the connectivity and coverage of the network severely, preventing the network from playing a proper role. The end-to-end connectivity refers to the correct transmission from one node to the final destination, which characterizes the ability of every node to report to the fusion center, thus it is important to ensure a high probability of connectivity [35]. Hence, the definition of network lifetime in this paper is consistent with references [7,14] and is defined as the time elapsed until the first sensor node in the network depletes its energy. We denote $e_{i}$ as the energy consumption of node $v_{i}$ in one round. $E_{i}$ is the total energy of node $v_{i}$. The formula of maximizing network lifetime Γ can be expressed as follows:
  (3) $$\max\left(\Gamma \right)=\min\underset{i\in \left\{1..m\right\}}{\max}\left(E_{i}/e_{i}\right)$$
(2) The scheme can locate attack sources quickly while defending against attacks.
  The spent time T for determining a malicious source is evaluated by the amount of marking information stored in attack paths. Obviously, in the process of reconstructing the attack paths, if the traceback scheme marks many data packets in the attack path, the system can collect much marking information quickly; then, the malicious node can be rapidly determined. Therefore, min(T) means to maximize marking information. ${𝒷}_{i}$ denotes the amount of marking information of node $v_{i}$ in a unit time; thus:
  (4) $$\min\left(T\right)=\underset{i\in \left\{0..m\right\}}{\max}\sum^{\text{​}}{𝒷}_{i}$$
(3) The average credibility of nodes, which is used to store marking tuples, is to be maximized.
  When the produced data packet is sent to the sink, the marking information of nodes can be added to the data packet. However, when the length of marking information reaches a certain value, the marking information can be stored in nodes. If marking tuples are stored in a malicious node, those marking tuples can be dropped or tampered with. Therefore, one goal of the TAPMS scheme is to maximize the average trust of nodes that store marking tuples. Consider that the trust of node $v_{i}$ is $c_{i}$. The number of marking tuples stored in node $v_{i}$ is $S_{i}$, as shown in Equation (5):
  (5) $$\text{max}\left(T\right)=\text{max}\left(\underset{i\in \left\{0..m\right\}}{\min}\sum^{\text{​}}\left(\;S_{i}{\;\text{c}}_{i}\right)/\underset{i\in \left\{0..m\right\}}{\min}\sum^{\text{​}}\left(\;S_{i}\right)\right)$$
  In summary, the optimization purpose of the scheme in this paper is:
  (6) $$\left\{ \begin{array}{c} \max\left(\Gamma \right)=\min\underset{i\in \left\{1..m\right\}}{\max}\left(E_{i}\right)\\ \min\left(T\right)=\underset{i\in \left\{0..m\right\}}{\max}\sum^{\text{​}}{𝒷}_{i}\\ \text{max}\left(T\right)=\text{max}\left(\underset{i\in \left\{0..m\right\}}{\min}\sum^{\text{​}}\left(\;S_{i}{\;\text{c}}_{i}\right)/\underset{i\in \left\{0..m\right\}}{\min}\sum^{\text{​}}\left(\;S_{i}\right)\right) \end{array}\right.$$

## 4. Trust-Based Adaptive Probability Marking and Storage Traceback Scheme

### 4.1. Research on Motivation

This study considers two factors: (1) the marking probability of nodes, the ability to detect malicious nodes and lifetime; (2) the security for storing marking tuples. Relevant parameters are listed in Table 2.

(1) In previous traceback schemes, after the system sets baseline marking probability (BMP) $P_{0}$, the marking probability is a constant $P_{0}$. The following problems result: (1) when the network is in a “safe” state, the probability that the system is attacked by malicious nodes is small. Setting low MP $P_{0}$ in this situation can reduce marking tuples routing in the path to improve network performance. However, when the network is not in a safe state, the system should adopt a high marking probability to determine the location of malicious sources in a very short period, thus contributing to network security.

**Figure 2:**
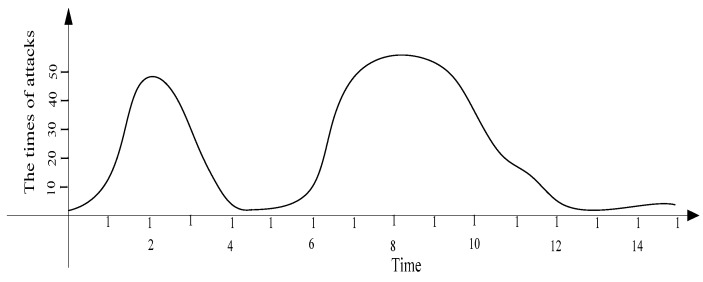
Attack times of the entire network.

Therefore, using a fixed MP cannot optimize network performance. An adaptive probability-marking scheme is proposed in this paper. For instance, the change in an attack behavior situation in different periods is provided in Figure 2. 

**Figure 3:**
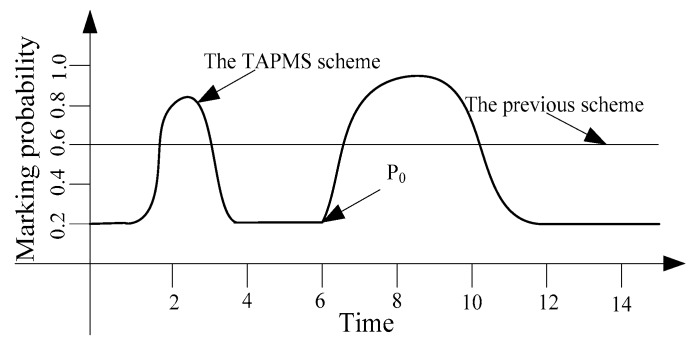
Marking probability in two schemes.

**Figure 4:**
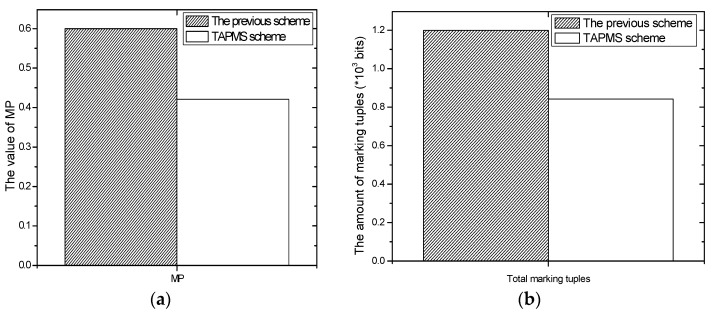
Average marking probability and the amount of marking tuples. (**a**) Average marking probability; (**b**) the amount of marking tuples.

Thus, the number of attacks is different at different times. Usually, when the attacks are sparse; it is unnecessary to adopt high a MP $P_{0}$ to avoid damage to the network lifetime. However, when the network is attacked by many malicious nodes, using a low MP $P_{0}$ cannot satisfy the network security requirement. TAPMS is used to improve network performance at a lower cost. The MP under different schemes is provided in Figure 3; a fixed MP $P_{0}$ is adopted in previous traceback schemes. However, the systems use different MP $P_{b}$ under different network security states in the TAPMS scheme. The average MP in the TAPMS scheme and other schemes with a fixed MP is provided in Figure 4. Because the network is in a safe state for most of the time, the average MP in the TAPMS scheme is lower than that in the other schemes; therefore, the total number of marking tuples in the TAPMS scheme is smaller than that in previous schemes (see Figure 4). Therefore, the TAPMS scheme can improve network lifetime.

**Figure 5:**
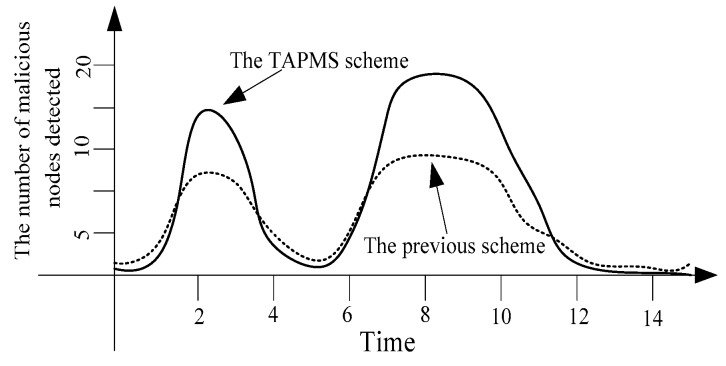
Number of malicious nodes detected.

The number of detected malicious nodes is provided in Figure 5. When the network is in a safe state, the number of malicious node in the network is smaller, so the number of detected malicious nodes is smaller. However, when the network is not in a safe state, the TAPMS scheme adopts a higher MP.

Thus, the probability of detecting malicious nodes is high, resulting in the total number of detected malicious nodes in the TAPMS scheme being greater than that in previous traceback schemes. Therefore, using the TAPMS scheme can improve network security.

(2) Security for storing marking tuples

**Figure 6:**
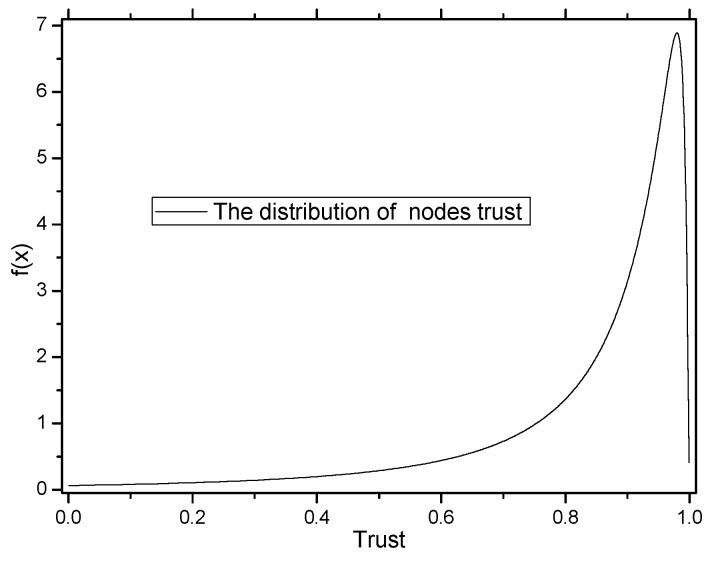
Distribution of node trust.

**Figure 7:**
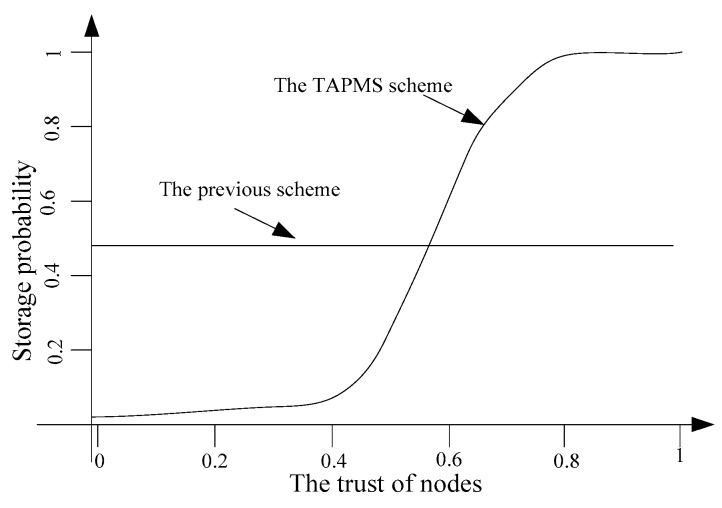
Number of malicious nodes detected.

**Figure 8:**
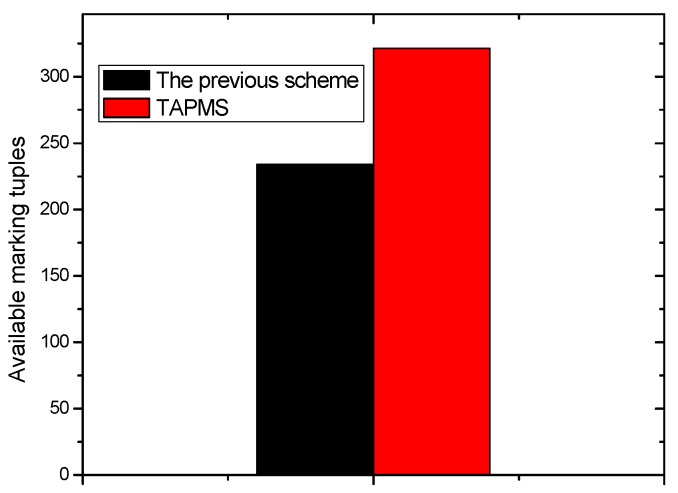
Available marking tuples.

The distribution of node trust in the network is shown in Figure 6. Many studies show that the distribution of node trust in a network is subject to a logarithmic normal distribution [4]. That is, the trust of most nodes in the network is high, which is consistent with the practice network [6]. Previous schemes do not focus on whether nodes that store marking tuples are safe. If marking tuples are stored in malicious nodes, those marking tuples can be dropped easily, resulting in traceback scheme failure. In the TAPMS scheme, most marking tuples are stored in high trust nodes. Thus, the security of storing marking tuples can be improved, thereby improving the effectiveness of the scheme. This improvement is shown in Figure 7. In previous schemes, the probabilities for storing marking tuples in all nodes are the same. In the TAPMS scheme, the probability of storing marking tuples in low trust nodes is low, and the probability of storing marking tuples in high trust nodes is high. The number of received available marking tuples after a period is provided in Figure 8. Figure 8 shows that the number of received available marking tuples in the TAPMS scheme is greater than that in other schemes, showing the effectiveness of the TAPMS scheme. There are two main differences in the TAPMS scheme compared with previous schemes: (1) the use of adaptive marking probability. Marking probability (MP) is low when network security is high but is large when network security is low.

This scheme can improve network security while reducing the number of transmitted marking tuples, thereby improving network lifetime; (2) nodes with high trust can be selected to store marking tuples to protect the validity of marking tuples. The TAPMS scheme is designed based on these two factors.

### 4.2. Trust-Based Adaptive Marking Probability Approach

In the TAPMS scheme, the marking probability is divided into two types: (1) when the network is safe, the system adopts a lower MP. This MP is called the baseline marking probability (BMP)${\;p}_{0}$; (2) When the network is not safe, that is, network security is in a bad situation, the system uses MP $p_{a}$. The marking probability can be calculated through obtaining the status of the network and then broadcast to each node in the network. The marking probability adopted in the network updates occurs every τ. Time slots can be denoted as $t≜\left\{𝓉={𝓉}_{0},{𝓉}_{1},{𝓉}_{2},\;\ldots ,{𝓉}_{n}\right\}$. Let $c_{i,j}$ denote the trust of node $v_{i}$ in time slot ${𝓉}_{j}$, which is evaluated by the sink. The trust of all nodes calculated in time slot ${𝓉}_{j}$ is as follows:

$$c_{j}=\sum_{i=1}^{m}c_{i,j}/m$$

The trust of nodes in the last w time slots can be calculated as follows:

$$C≜\left\{c_{1},c_{2},\;\ldots ,c_{w}\right\}$$

Then, the average trust of the network can be calculated as follows:

(7) $$C_{w}=\{\begin{array}{c} \sum_{k=1}^{w}\;c_{k}\times 𝒽\left(k\right)/w,\;w\neq 0\\ 1,\;w=0 \end{array}$$

where 𝒽(k) is an attenuation function that can be shown as follows:

(8) $$𝒽\left(k\right)=\{\begin{array}{c} 1,\;k=w\\ 𝒽\left(k-1\right)=𝒽\left(k\right)-1/w,\;1\leq k\leq w \end{array}$$

When the system calculates the network’s trust, it considers the most recent w time’s evaluation results. 𝒽(k) ensures that the recent trust evaluation results have a higher weight [6]. In the TAPMS scheme, the marking probability of the network is calculated according to the network trust. The marking probability function has the following properties: when the network’s trust is above a certain threshold, the network adopts baseline marking-probability (BMP)$P_{0}$; otherwise, the system adopts a high marking probability according to the network’s trust. In the TAPMS scheme, the conversion function from the network’s trust to the marking probability is as follows:

(9) $$ℒ\left(C\right)=\{\begin{array}{c} \left(\frac{1}{\sin\left(C_{1}\right)}-\frac{1}{\sin\left(C_{0}\right)}\right)/𝕒+\left(C_{1}-C_{0}\right)\varepsilon ,\;if\;C<c_{1}\\ p_{0}\;,\;else \end{array}$$

where $C_{0}$ is the baseline trust, $C_{h}$ is the max trust, and C is the result of the network’s trust evaluation at the current time; $𝕒=\left(\frac{1}{\sin\left(C_{h}\right)}-\frac{1}{\sin\left(C_{0}\right)}\right)$:

(10) $$p_{0}=\left(\frac{1}{\sin\left(C_{1}\right)}-\frac{1}{\sin\left(C_{0}\right)}\right)/𝕒+\left(C_{1}-C_{0}\right)\varepsilon$$

The major function of the marking probability controller is to adjust the marking probability of the network, which makes the probability equal to the probability calculated by Equation (9). Considering that the trust evaluation result of the network in time slot ${𝓉}_{j}\;$is $C_{j}$, the calculated marking probability based on Equation (9) is $ℒ\left(C_{j}\right)$. In fact, the marking probability of the current network is $P_{j}$; if $ℒ(C_{j})>P_{j}$, the system can increase the marking probability of the network. Otherwise, the system decreases the marking probability. In the TAPMS scheme, the adjustment function of the marking probability controller is a simple dynamic model; the change rate of the marking probability of the network is directly proportional to the gradient of the utility function:

(11) $$\frac{\partial ℒ\left(C\right)}{\partial C}=\left(\frac{\csc\left(C\right)}{{\left(\sin\left(C\right)\right)}^{2}}\right)/𝕒$$

The characteristics of the utility functions guarantee that there is an optimum value [6]. In the time interval between the current time ${𝓉}_{j}$ and the next period ${𝓉}_{j+1}$, the iterative equation of the marking probability of the network can be expressed as follows:

(12) $$P_{j+1}=P_{j}+{𝕧}_{i}\frac{\partial ℒ\left(C\right)}{\partial C}$$

where ${𝕧}_{i}$ > 0 indicates the adjustment step of the marking probability of the network; therefore, the marking probability of the network in the next period can be expressed as follows:

(13) $$P_{j+1}=\{\begin{array}{c} p_{0}\;if\;c\geq c_{0}\\ P_{j}+{𝕧}_{i}\left(\frac{\csc\left(C\right)}{{\left(\sin\left(C\right)\right)}^{2}}\right)/𝕒,else \end{array}$$

When the difference between the measured trust and the adopted trust of the marking probability of the network exceeds predetermined threshold ∆, the sink node notifies the nodes that its marking probability should be updated by Equation (13). Therefore, the average marking probability of the entire network is as follows:

(14) $$\bar{P}=\int_{c_{0}}^{c_{h}}ℒ\left(C\right)f\left(c\right)$$

f(c) is the distribution function of the network’s trust. As proved in [16], the amount of data assumed by the nodes at l = hr+x away from the sink is as follows:

(15) $$d_{l}=\left(\left(z+1\right)+\frac{z\left(z+1\right)r}{2l}\right)\lambda \;|z=⎣\frac{R-l}{r}⎦$$

The number of total marking tuples is as follows:

(16) $$𝕞=\int_{0}^{R}\bar{P}2\pi x\rho d_{l}d_{x}=2\pi \bar{P}\rho \int_{0}^{R}xd_{l}d_{x}$$

where ρ is the node density. From Equation (16), the 𝕞 in different traceback schemes is only related to $\bar{P}$. The value of $\bar{P}$ depends of f(c) and ℒ(c); f(c) is the node trust-distribution function and is determined by physical properties of the network. The important parameters in ℒ(c) depend on the value of $P_{0}$. The following discusses how to select optimized $P_{0}$. In this paper, many conclusions are obtained; the utility function between obtained payoff and the number of marking tuples (cost) is a non-linear function [6]. The non-linear function is consistent with the characteristics of the network. That is, when the number of collected marking tuples is small, payoff rises quickly as the number of collected marking tuples increases; therefore, the payoff’s utility is high. However, when the number of collected marking tuples reaches a certain point, the system increases the number of collected marking tuples; the growth of the payoff is very small, and its utility is small. Following Ref. [6], Equation (17) is adopted as a utility function for TAPMS:

(17) ℱ(𝕞)=αlog(1+𝕞)+β𝕞

where α, β are constant parameters. The obtained payoff is the difference between utility function and cost. The system pays an energy consumption price to send marking tuples; the cost can be obtained from Equations (1) and (2). The paid cost is a linear relationship with the number of sending marking tuples, namely, γ𝕞,γ, which are constant coefficients. The payoff function of this system can be expressed as follows:

(18) K=αlog(1+𝕞)+β𝕞−γ𝕞

**Theorem 1.**  *In the TAPMS scheme, the optimal number of marking tuples* 𝕞 *to maximize its payoff is provided in Equation (19);* ${𝕞}^{*}$ *is an optimal value*:

(19) $${𝕞}^{*}=\frac{\alpha }{\beta -\gamma }-1$$

**Proof.**  Obviously, 𝕞 in the system is a bounded closed set in Euclidean space. The payoff function Equation (18) of the system is continuous on the strategy space [6]. The following proves that the payoff function is a concave function; therefore, there is an optimum value. The first- and second-order derivatives of Equation (18) with respect to 𝕞 are as follows:

$$\frac{\partial K}{\partial \left(𝕞\right)}=\frac{\alpha }{1+𝕞}+\beta -\gamma$$

$$\frac{\partial^{2}K}{\partial^{2}\left(𝕞\right)}=-\frac{\alpha }{{\left(1+𝕞\right)}^{2}}<0$$

Because $\frac{\partial^{2}K}{\partial^{2}\left(𝕞\right)}<0$, A is strictly concave in 𝕞. Hence, the optimal 𝕞 that maximizes K is determined by letting the marginal utility $\frac{\partial K}{\partial \left(𝕞\right)}$ be equal to 0, *i.e.*,$\;\beta -\gamma =\frac{\alpha }{1+𝕞}$. $𝕞=\frac{\alpha }{\beta -\gamma }-1$, which leads to Equation (19).

Based on optimized ${𝕞}^{*}$ and on Equations (9), (14) and (16), the optimized baseline marking probability (BMP) $P^{0}$ can be obtained. The Algorithm 1 of the TAPMS scheme is provided. First, all the network trusts are 0.5; as the network operates, the system can increase or decrease its marking probability for different nodes depending on the node trust to achieve an optimal probability.

**Algorithm 1:** The adaptive probability marking traceback approach**Initialize:** Let the network’s trust be 0.5;1:**For** each time t **Do**2: Compute 𝕞 according to Equation (19);3: Compute $p_{0}$ according to Equations (9), (14) and (16);4: Compute the network’s trust;5: Compute the marking probability by Equation (9) of the node;6: Compute each node’s $P_{i+1}$ by Equation (13);7: Broadcast each node’s $P_{i+1}$;8: t←t+1; //next time t9:**End For**

### 4.3. Trusted Storage Approach

The main innovation of the TAPMS scheme is that the marking tuples are stored in nodes with high trust to make stored marking tuples more credible, which can enhance the effectiveness of the network. In the traceback scheme, marking tuples are stored in nodes by logging; therefore, this goal can be reached through adjusting the logging probability. The specific method is that high trust nodes adopt a high logging probability, whereas low trust nodes adopt a low logging probability. In this paper, we assume that node trust can be obtained through monitoring the network (including the traceback scheme). After obtaining node trust, it is possible to implement the proposed TAPMS scheme. The TAPMS scheme uses a simple trust-grading logging method which can be seen from Algorithm 2. The method is that the logging probability of nodes is 0 when node trust is below a certain threshold $c_{s}$, and nodes adopt low logging probability when node trust is in $\left[c_{s},\;c_{z}\right]$. However, nodes adopt a high logging probability when node trust >$c_{z}$. Marking tuples can be stored in high trust nodes through the above method, thereby enhancing the effectiveness of the traceback scheme.

**Algorithm 2:** The adaptive logging approach 1:**For** each time t **Do**2: The trust of each node $\{c_{1,t,}\;c_{2,t},\;c_{3,t\;},\;\ldots ,\;c_{m,t}\}\;$at time t is computed by the Sink;3: **For** each node $v_{i}$ **Do**4: **Case** $c_{i,t}$5: $\left[0,\;c_{s}\right]:\;S\left(v_{i}\right)=0;$ //$\;S\left(v_{i}\right)$ is the logging probability of $v_{i}$6: $\left[c_{s},\;c_{z}\right]:\;S\left(v_{i}\right)=\varphi x;$ // φ ∈(0,1) is a constant7: $\left[c_{z},\;1\right]:\;S\left(v_{i}\right)=\min\left(\phi x,1\right)\;$ //ϕ>1 is a constant8: **End For**;9: **For** each packet $B_{i}$ received by node $v_{i}$ **Do**;10: node $v_{i}$ logs marking tuples of packets $B_{i}$ with probability $S\left(v_{i}\right)$;11: **End For**;12: t←t+1; //next time t13:**End For**

## 5. Performance Analysis and Optimization

This section analyzes the performance of the TAPMS scheme. The performance of the TAPMS scheme primarily includes three parts: (1) Lifetime. The energy consumption is different in different schemes, so the lifetime is also different; (2) Probability of detecting malicious nodes. If the ability to find malicious nodes is stronger in the traceback scheme, the probability of finding malicious nodes is greater; thus, most malicious nodes can be eliminated to ensure the security of the network. The probability of finding a malicious node can be used as an important indicator to express the effectiveness of the scheme; (3) Security for storing marking tuples.

### 5.1. Energy Consumption and Network Lifetime

**Figure 9:**
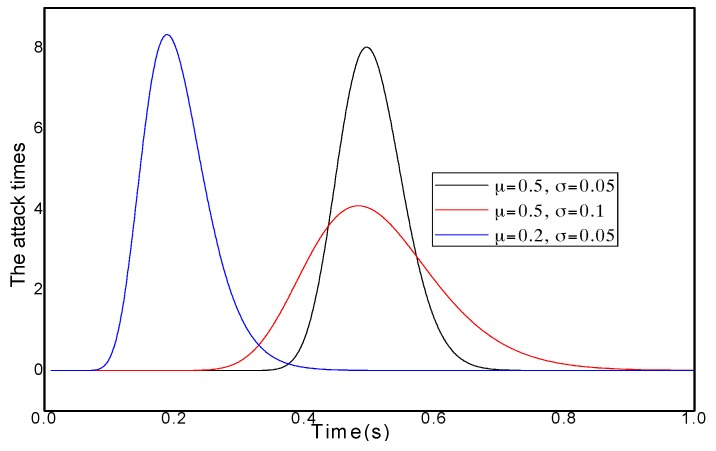
Density distribution of attack times of malicious nodes.

For a determined wireless sensor network, the energy consumption of a node is primarily composed of two parts. One part is the energy consumption for forwarding data. The other part is the energy consumption for forwarding marking tuples. For different traceback schemes, the energy consumption for forwarding data is the same. Therefore, analyzing the number of marking tuples in different schemes is equivalent to analyzing energy consumption in different schemes. To analyze the number of transmitting marking tuples in different traceback schemes, we assume that the attack times of malicious nodes obey a logarithmic normal distribution [36]. In other words, the attack times of malicious nodes are sparse most of the time (see Figure 9). The logarithmic normal distribution function is shown as Equation (20):

(20) $$f\left(x\right)=\{\begin{array}{c} \frac{1}{x\sigma \sqrt{2\pi }}e^{-\frac{{\left(lnx-\mu \right)}^{2}}{2\sigma^{2}}}\;,\;x>0\\ 0\;,else \end{array}$$

The feature density of the logarithmic normal distribution is concentrated in the vicinity of the expectation value, whereas the density in other areas is small. Thus, the function is suited to describing a network in which the time of an attack launched by malicious nodes is usually short. μ determines the concentration degree of attack times; μ being larger shows that the attack behavior is concentrated in a small period.

Considering that the marking probability in the MP scheme is $P_{mp}$, according to [37], node $v_{k}$, whose distance from the sink is l m, forwards data packets from nodes whose distance from the sink is l, l+r, l+2r, l+3r,…,l+zr | z=⎣(R−l)/r⎦. For data packets from nodes whose distance from the sink is l+ir, a marking tuple can be added to the data packet at probability $P_{mp}$ when a data packet is transmitted from one node to another node. The data packet must be transmitted i hops from nodes whose distance from the sink is l+ir to node $v_{k}$; then, node $v_{k}$ forwards the data packet to the next hop. Therefore, the number of marking tuples is $(i+1)P_{mp}$.

The number of data packets forwarded from nodes whose distance from the sink is l+ir by node $v_{k}$, whose distance from the sink is l, is (l+ir)/l. Therefore, the total number of forwarding marking tuples is $\left(i+1\right)P_{mp}\left(l+ir\right)/l$. The total number of forwarding marking tuples of node $v_{k}$ is shown as Equation (21):

(21) $$\begin{array}{c} m_{l,mp}=P_{mp}+2P_{mp}(l+r)/l+3P_{mp}(l+2r)/l+,\ldots ,(z+1)P_{mp}(l+zr)/l\\ =P_{mp}\sum_{i=0}^{z}\{(z+1)(l+zr)/l\} \end{array}$$

In the TAPMS scheme, the average marking probability of each node is Equation (22):

(22) $$P_{tapms}=\int_{0}^{1}ℒ\left(C\right)N\left(f\left(x\right)\right)dx$$

In Equation (23), N(f(x)) is the function used to convert the attack density function of a node to the density function of network trust. ℒ(C) is the function used to convert trust to marking probability. Obviously, the number of marking tuples forwarded by node $v_{k}$ is given by Equation (23):

(23) $$m_{l,tapms}=P_{tapms}\sum_{i=0}^{z}\{(z+1)(l+zr)/l\}$$

because network lifetime depends on the lifetime of the node with maximum energy consumption in the network, that is, $\ell =E_{init}/E_{max}$. The energy consumption of a node includes the energy consumption for forwarding data and the energy consumption for forwarding marking tuples. According to Equation (15), the number of forwarded data packets of the node whose distance from the sink is l m is $d_{l}$. Because the energy consumption of the node nearest the Sink is maximum, set the distance of the node nearest the sink to $l_{min}$. Therefore, network lifetime in the MP scheme can be expressed as follows:

(24) $$\ell_{mp}=E_{init}/\left(e_{pa}d_{l_{min}}+e_{ma}m_{l_{min},mp}\right)$$

where $e_{pa}$ and $e_{ma}$ represent energy consumption for forwarding a data packet and a marking tuple, respectively.

Network lifetime in the TAPMS scheme can be expressed as follows:

(25) $$\ell_{tapms}=E_{init}/\left(e_{pa}d_{l_{min}}+e_{ma}m_{l_{min},tapms}\right)$$

In the two schemes represented by Equations (24) and (25), the marking probabilities $P_{mp}$ and $P_{tamps}$ are different. If the network is in a safe state most of the time, the average marking probability $P_{tamps}$ < $P_{mp}$; therefore, the network lifetime in the TAPMS scheme is greater than that in previous schemes. Obviously, if the attack times in the network are greater, the network lifetime in TAPMS is the same as the network lifetime in other schemes. Conversely, the marking probability affects not only the network lifetime but also the effectiveness of the security. Thus, reducing the marking probability to a certain value can affect network security. Therefore, the safety effectiveness of the TAPMS scheme is analyzed theoretically in the next section.

### 5.2. Detection Probability Analysis

Recognizing that the probability of malicious nodes is a linear relationship with MP, a higher MP is associated with a higher probability of identifying malicious nodes. At the same time, the probability of identifying malicious nodes is also associated with attack frequency. More-frequent attacks in the network are associated with a higher probability of identifying malicious nodes. As mentioned previously, the network security state can be described by a logarithmic normal distribution. Thus, if MP is $P_{mp}$, the expectations value for detecting malicious nodes is as follows:

(26) $$J_{\mathrm{mp}}=\int_{0}^{1}P_{mp}𝓊𝓋yf(y)=\rho 𝓊𝓋\int_{0}^{1}y\frac{1}{y\sigma \sqrt{2\pi }}e^{-\frac{{\left(lny-\mu \right)}^{2}}{2\sigma^{2}}}dy=\rho 𝓊𝓋e^{\left(\mu +\sigma^{2}/2\right)}$$

where 𝓊 is an adjustment factor that is used to convert MP $P_{mp}$ to a recognition rate, and 𝓋 is a coefficient between attack times and the probability of finding malicious nodes.

The probability of identifying malicious nodes in TAPMS is as follows:

(27) $$J_{\text{tapms}}=\int_{0}^{1}ℒ\left(C\right){𝓊}_{1}𝓋yf\left(y\right)dy$$

### 5.3. Average Trust of Storage

**Figure 10:**
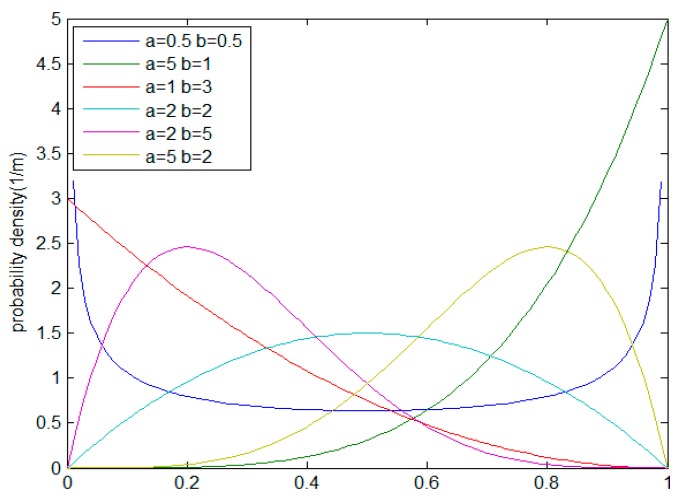
Distribution of the Beta function.

Another important difference of the TAPMS scheme compared with previous schemes is that marking tuples are stored on nodes with high trust. Consider that node trust obeys a beta distribution [38]. The mathematical form of the Beta distribution is shown as Equation (28); the variable x ranges from 0–1. Therefore, *x* can describe node trust in the range (0, 1). When a and b in the Beta distribution are greater than 0, the two parameters determine the shape of the distribution function, as shown in Figure 10:

(28) $$f\left(x\right)=\{\begin{array}{c} \frac{1}{B\left(a,b\right)}x^{a-1}{\left(1-x\right)}^{b-1}\;,\;0<x<1\\ 0\;,else \end{array}$$

where $B\left(a,b\right)=\int_{0}^{1}x^{a-1}{\left(1-x\right)}^{b-1}dx$.

The expectations value of the Beta distribution is shown as Equation (29):

(29) $$E\left(X\right)=\int_{0}^{1}\frac{x}{B\left(a,b\right)}x^{a-1}{\left(1-x\right)}^{b-1}dx=\frac{1}{B\left(a,b\right)}\int_{0}^{1}x^{a}{\left(1-x\right)}^{b-1}dx=\frac{B\left(a+1,b\right)}{B\left(a,b\right)}=\frac{a}{a+b}$$

**Figure 11:**
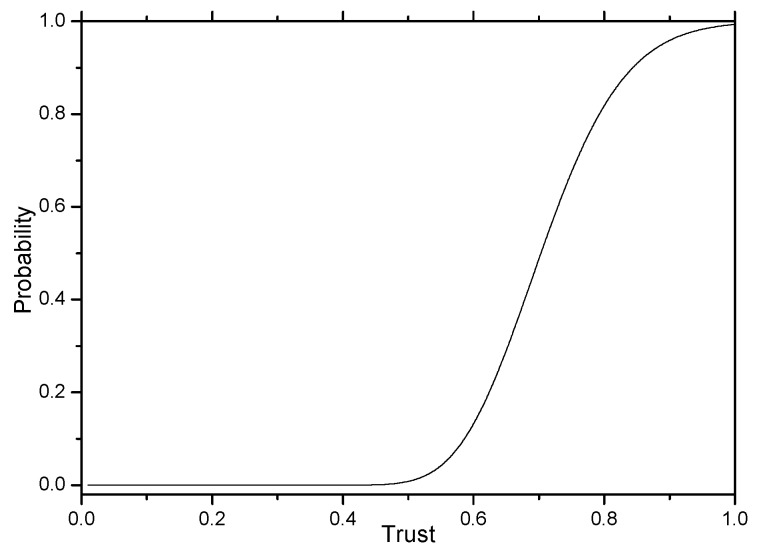
Distribution function of node trust.

In a Beta distribution function, if the value of a is larger and the value of b is smaller, the Beta function can be used to express the distribution of node trust. That is, the trust of most nodes is high, so the average trust of a node is greater than 0.5. The distribution function of node trust is shown in Figure 11.

Nodes with different trust have different reliabilities for storing marking tuples because trust and reliability satisfy the positive correlation functions. Because ${ℛ}_{i}$ is the reliability of node $v_{i}$, the trust of node $v_{i}$ is $c_{i}$, and γ is a constant, we have the following:

(30) $${ℛ}_{i}=\;\gamma c_{i}$$

Thus, if the system adopts a conventional storage scheme, namely, that marking tuples are randomly stored on any node, then the average trust of nodes that store marking tuples under this scheme is as follows:

(31) $$E\left(S_{1}\right)=\int_{0}^{1}\frac{\gamma x}{B\left(a,b\right)}x^{a-1}{\left(1-x\right)}^{b-1}dx=\gamma \frac{a}{a+b}$$

If the system adopts the method in which nodes whose trust is less than threshold $c_{s}$ do not store marking tuples, the average trust of nodes that store marking tuples is as follows:

(32) $$\int_{c_{s}}^{1}\gamma xf\left(x\right)dx=\gamma \frac{a}{a+b}-\int_{0}^{c_{s}}\gamma xf\left(x\right)dx$$

In the TAPMS scheme, nodes with high trust adopt a high probability of storing marking tuples. Because the probability of storing marking tuples is S(x) with x representing node trust, using piecewise functions to express node trust can be shown as follows:

(33) $$S\left(x\right)=\{\begin{array}{c} 0\;,\;0<x<c_{s}\\ \varphi x\;,\;c_{s}\leq x<c_{z},\;0<\varphi <1\\ \min\left(\phi x,1\right)\;,\;c_{z}\leq x<1,\;1<\varphi <3 \end{array}$$

The average node trust is as follows:

(34) $$E\left(S_{2}\right)=\int_{0}^{1}S\left(x\right)f\left(x\right)dx=\int_{c_{s}}^{c_{z}}\varphi xf\left(x\right)dx+\int_{c_{z}}^{1}\min\left(\phi x,1\right)f\left(x\right)dx$$

If nodes whose trust is less than $c_{s}$ do not store marking tuples, only $c_{z}$ is variable in Equation (34). If the storage reliability is A, then $c_{z}$ can satisfy equation Equation (35):

(35) $$A=\int_{c_{s}}^{c_{z}}\varphi xf\left(x\right)dx+\int_{c_{z}}^{1}\min\left(\phi x,1\right)f\left(x\right)dx$$

## 6. Experimental Results

OMNET++ is used for experimental verification [39]. In the experiment, the setup is as follows: the network radius *R* = 400, there are 1000 nodes in the network, and there are 100 malicious nodes.

### 6.1. Marking Probability and the Number of Receiving and Sending Marking Tuples

**Figure 12:**
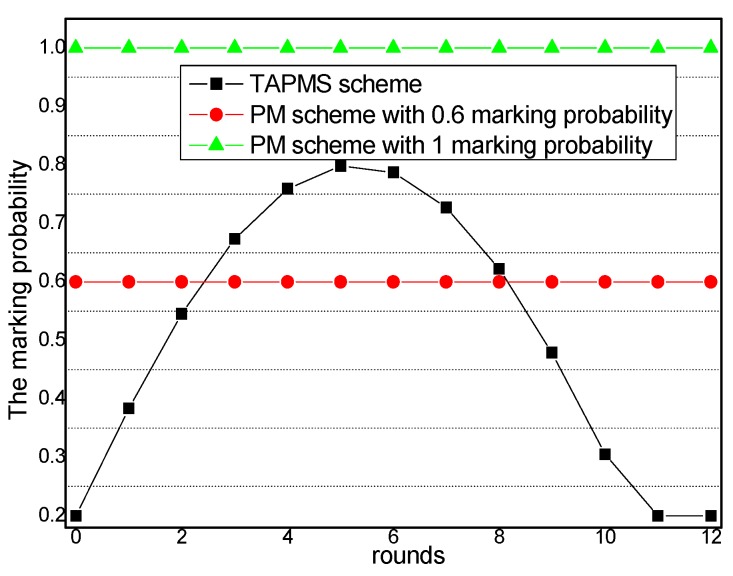
Marking probability.

The marking probability of nodes in the network under different network security states is provided in Figure 12. When the network is in a safe state, the marking probability of nodes in the TAPMS scheme is lower than that in other schemes; thus, the number of marking tuples that is transmitted to the sink is smaller. The energy consumption in the TAPMS scheme at this time is less than that in other schemes; therefore, network lifetime can be improved.

**Figure 13:**
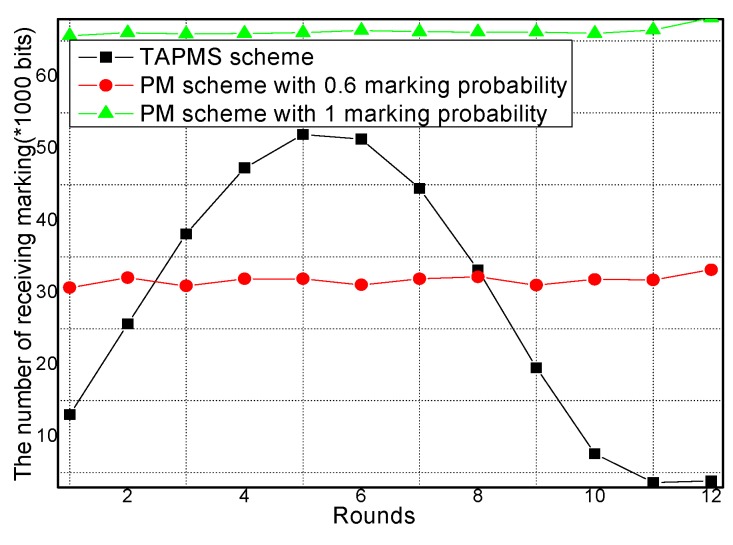
Amount of receiving marking information.

**Figure 14:**
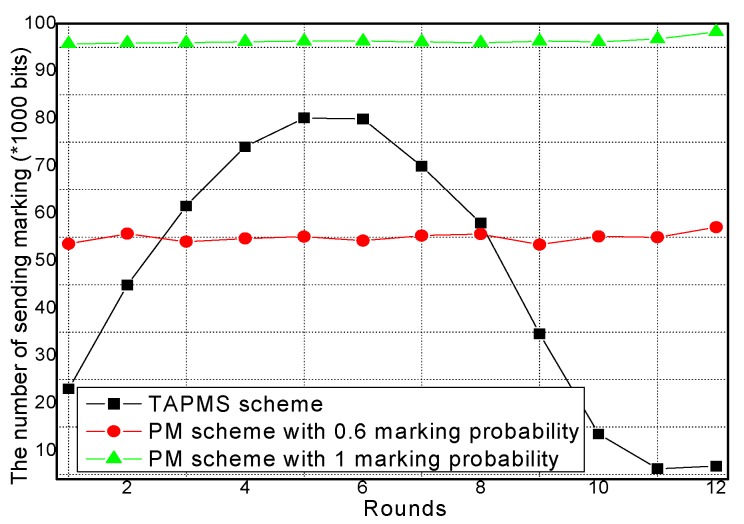
Amount of sending marking information.

The amounts of receiving marking information and sending marking information under different network security states are provided in Figure 13 and Figure 14, respectively. From Figure 13 and Figure 14, conclusions can be drawn that when the network is in a safe state, the number of receiving and sending marking tuples in TAPMS is smaller than that in other schemes. However, when the network is not in a safe state, the number of receiving and sending marking tuples in the TAPMS scheme is greater than that in a PM scheme with 0.6 marking probability. 

**Figure 15:**
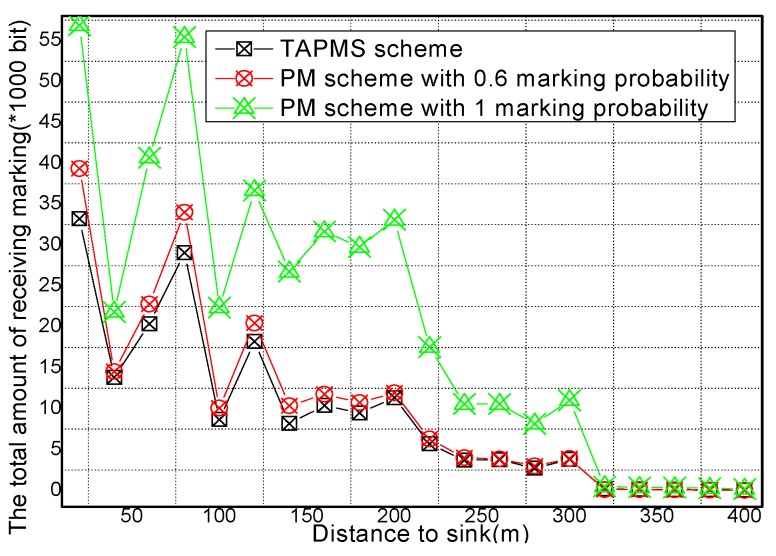
Total amount of receiving marking information under different distances to the Sink.

**Figure 16:**
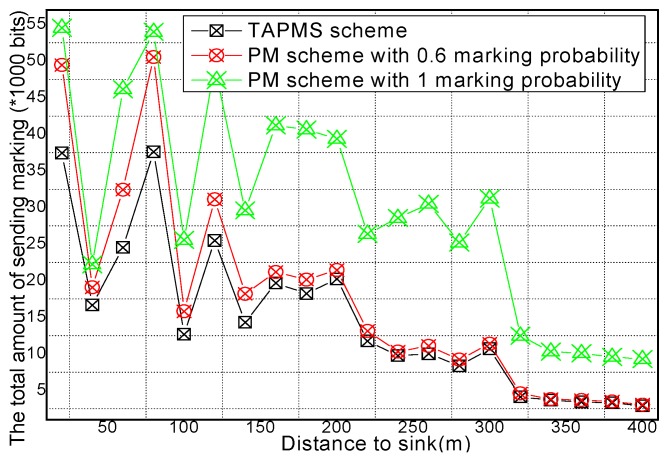
Total amount of sending marking information under different distances to the Sink.

The reason is that in the TAPMS scheme, the marking probability of nodes in the network is increased when the network is not in a safe state, but the marking probability of a node in the network is decreased when the network is in a safe state. The total amount of receiving marking information and sending marking information are provided in Figure 15 and Figure 16, respectively. Figure 15 and Figure 16 show that the total amount of receiving marking information and sending marking information in the TAPMS scheme is less than that in other schemes. Although the amount of receiving and sending marking information in the TAPMS scheme is less than that in other schemes when the network is in a safe state, the amount of receiving and sending marking information in the TAPMS scheme is greater than that in other schemes when the network is not in a safe state. 

However, the total amount of receiving marking information and sending marking information in the TAPMS scheme remains less than that in other schemes, which can improve network lifetime.

### 6.2. Number of Stored Marking Tuples and Energy Consumption

**Figure 17:**
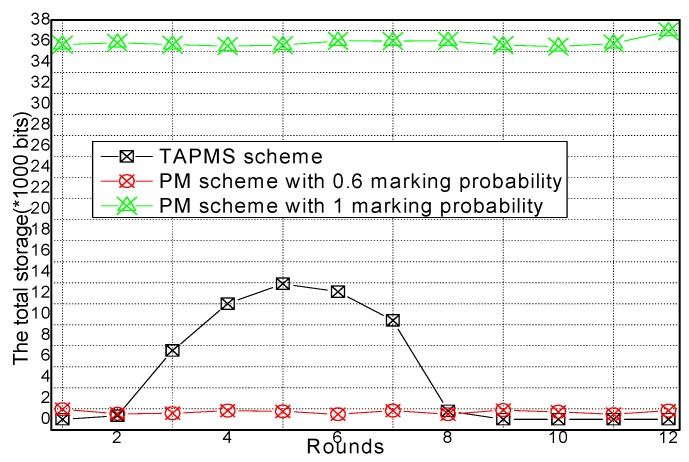
Total storage space under different network security states.

**Figure 18:**
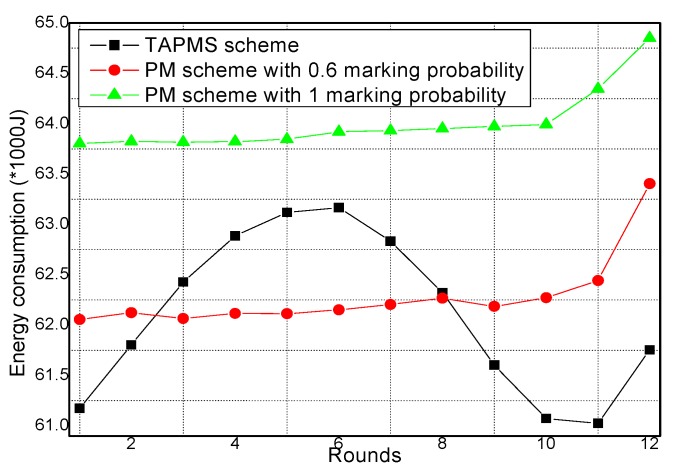
Energy consumption under different network security states.

The total amounts of stored marking information and energy consumption under different network security states are provided in Figure 17 and Figure 18, respectively. Figure 17 shows that when the network is in a safe state, the total amount of stored marking information in TAPMS is less than that in other schemes. However, when the network is not in a safe state, the total amount of stored marking information in TAPMS is greater than that in a PM scheme with 0.6 marking probability. The reason is the same as stated previously. Figure 18 shows that when the network is in a safe state, due to the low marking probability of nodes in the network, the total amount of stored marking information in TAPMS is less than that in other schemes. Therefore, the energy consumption in the TAPMS scheme is less than that in other schemes. However, when the network is not in a safe state, due to the higher marking probability of a node in the network, the total amount of stored marking information in TAPMS is greater than that in a PM scheme with 0.6 marking probability. Therefore, the energy consumption in the TAPMS scheme is greater than that in a PM scheme with 0.6 marking probability.

**Figure 19:**
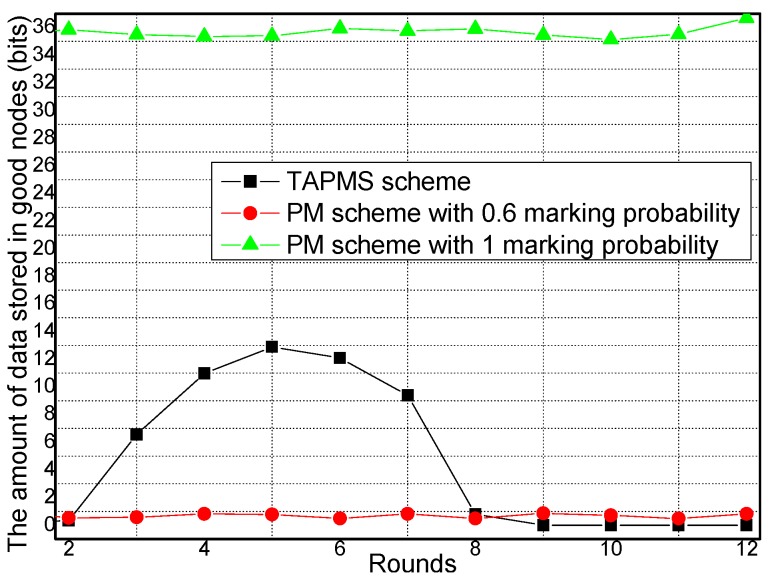
Amount of data stored in good nodes under different network security states.

**Figure 20:**
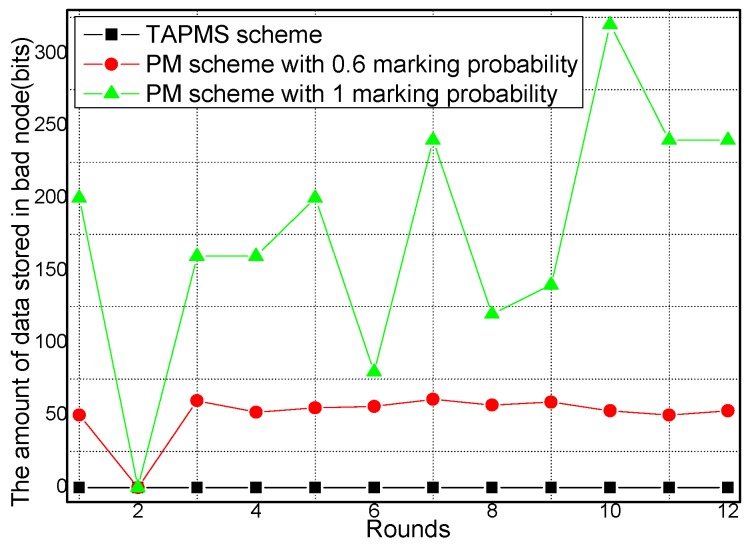
Amount of data stored in malicious nodes under different network security states.

The amounts of data stored in good nodes and malicious nodes under different network security states are provided in Figure 19 and Figure 20, respectively. Figure 19 shows that when the network is not in a safe state, the amount of data stored in the good node sin the TAPMS scheme is greater than that in the PM scheme with 0.6 marking probability because in the TAPMS scheme, nodes with high trust are selected to store marking tuples. Thus, the majority of marking tuples are stored in the good nodes, which can avoid the problem of marking information loss. Figure 20 shows that the amount of data stored in malicious nodes in the TAPMS scheme is greater than in the PM scheme. Thus, the performance of the TAPMS scheme is effective.

**Figure 21:**
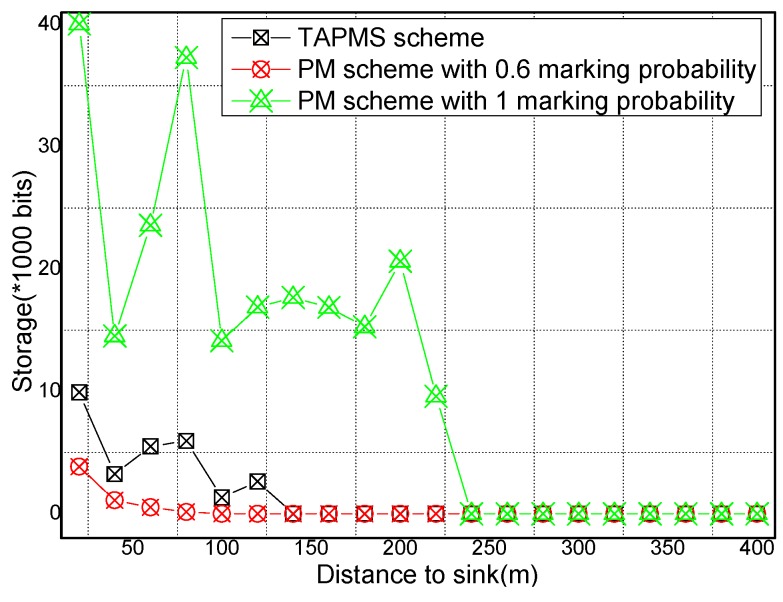
Storage space of nodes under different distances to the sink.

**Figure 22:**
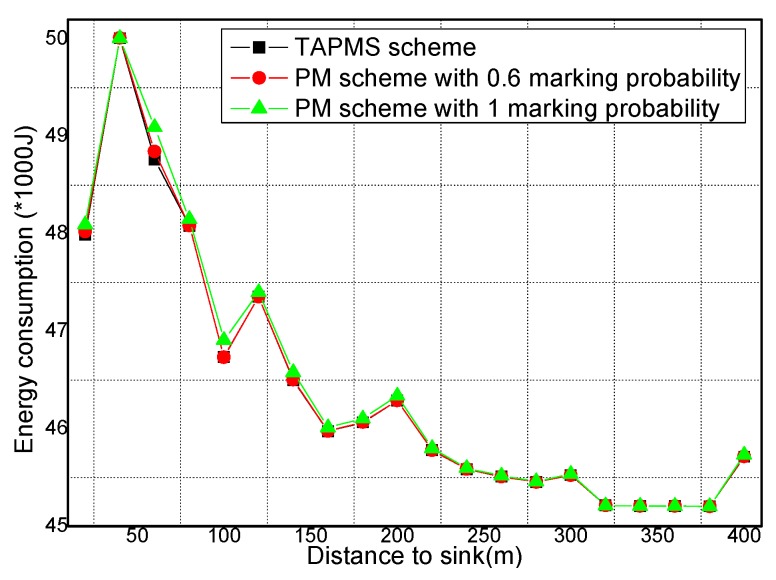
Energy consumption of nodes under different distances to the sink.

The storage spaces for node and energy consumption of nodes at different distances to the Sink are provided in Figure 21 and Figure 22, respectively. Figure 21 shows that for nodes in the range of 150 m to the sink, the storage space of those nodes in the TAPMS scheme is greater than that in the PM scheme with 0.6 marking probability, which shows that the malicious source node can be determined quickly when the network is attacked by a malicious node. Figure 22 shows that the energy consumption of a node in the TAPMS scheme is the same as the energy consumption in other schemes. Figure 18 shows that the energy consumption of the network in the TAPMS scheme is greater than that in the PM scheme with 0.6 marking probability when the network is not in a safe state. The total energy consumption in the TAPMS scheme ranges up to that in other schemes. Therefore, network lifetime in the TAPMS scheme cannot be damaged.

### 6.3. Security and Lifetime Performance

**Figure 23:**
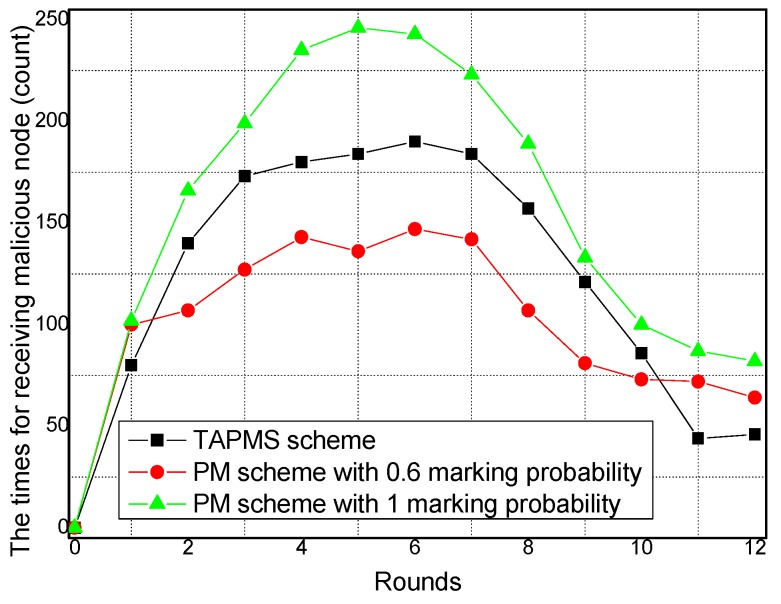
Times for receiving malicious nodes under different network security states.

The times (or the marking tuples) for receiving malicious nodes under different network security states are provided in Figure 23. When the network is in a safe state, that is, in rounds 1, 11, and 12, the time for receiving a malicious node in the TAPMS scheme is less than that in other schemes. However, the time for receiving a malicious node in the TAPMS scheme is greater than that in the PM scheme with 0.6 marking the probability when the network is not in a safe state. The reason is that when the network is not in a safe state, the number of attacks launched by malicious nodes is increased. To determine the malicious source node as soon as possible, the marking probability of a node can be increased in the TAPMS scheme. Therefore, the number of receiving malicious nodes is increased. However, when the network is in a safe state, the number of attacks launched by malicious nodes is smaller. The marking probability of a node can be decreased to save energy, showing that the TAPMS scheme can adjust the marking probability with the change of the network security state. The system can determine the malicious source node quickly using the TAPMS scheme when the network is not in a safe state, illustrating the effectiveness of the TAPMS scheme.

**Figure 24:**
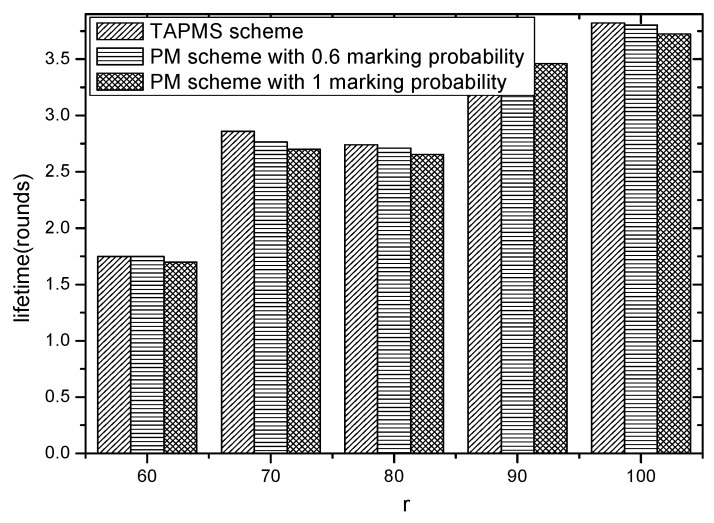
Network lifetime under different *r.*

**Figure 25:**
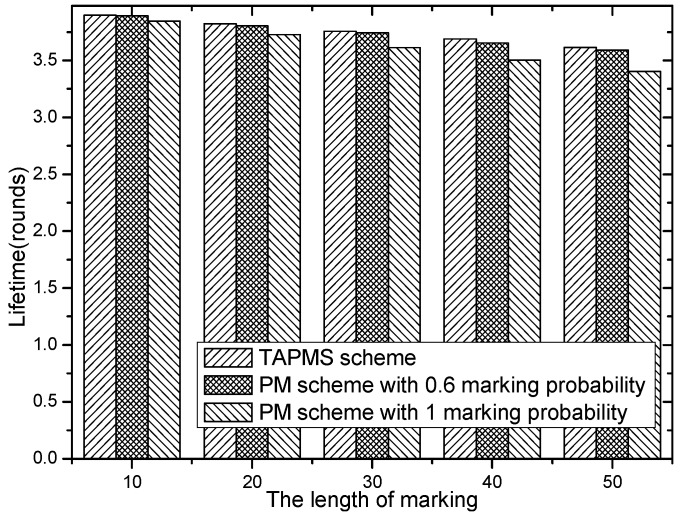
Network lifetime under different lengths of marking information.

Network lifetimes under different r and different lengths of marking information are given in Figure 24 and Figure 25. Figure 24 shows that network lifetime in the TAPMS scheme is greater than that in other schemes. Figure 25 implies that: (1) a longer length of marking information implies a smaller network lifetime and (2) network lifetime in the TAPMS scheme is greater than that in other schemes.

The reason is that in the TAPMS scheme, the marking probability of a node in the network is adjusted with the change of the network security state. The marking probability of a node becomes large to improve the ability to resist attacks only when the network state is not in a safe state. Usually, the network is in a safe state, and the marking probability of a node is smaller in the TAPMS scheme than in other schemes. Therefore, network lifetime is greater in the TAPMS scheme than in other schemes.

## 7. Conclusions

In this paper, we have proposed an adaptive probability marking traceback (TAPMS) scheme for reducing traceback time and enhancing network lifetime. The TAPMS scheme adopts an adaptive control mechanism to adjust the marking probability of a node according to the security requirement of the network. When the network is in a safe state, the marking probability of a node is high, but the marking probability of a node in the network is low when the network is not in a safe state. Therefore, network lifetime can be improved effectively. The most important point is that the marking tuples should be stored in the nodes with high trust to avoid stored marking tuple loss. To do this, the malicious nodes can be backtraced quickly, and the total number of marking tuples is also smaller than in other schemes. Thus, the lifetime and traceback time performance can be enhanced. 

## Figures and Tables

**Table 1:** Network parameters.

Parameter	Value
Threshold distance (*d*_0_) (m)	87
Sensing range *r_s_* (m)	15
*E_elec_* (nJ/bit)	50
ε*_fs_* (pJ/bit/m^2^)	10
ε*_amp_* (pJ/bit/m^4^)	0.0013
Initial energy (J)	0.5

**Table 2:** Parameter description.

Parameter	State
$P_{0}$	Baseline marking probability (BMP)
$c_{i,j}$	Trust of node $v_{i}$ in time slot ${𝓉}_{j}$
$C_{w}$	Average trust of network in the last w time
$C_{0}$	Baseline trust
$C_{h}$	Max trust
$\bar{P}$	Average marking probability of the entire network
𝕞	Number of marking tuples
${𝕞}^{*}$	Optimal value of 𝕞 to maximize its payoff
$c_{s}$	Trust threshold
${ℛ}_{i}$	Reliability of node $v_{i}$
$c_{i}$	Trust of node $v_{i}$
γ	Constant
